# The Effects of Arousal and Approach Motivated Positive Affect on Cognitive Control. An ERP Study

**DOI:** 10.3389/fnhum.2018.00320

**Published:** 2018-08-31

**Authors:** Andrzej Cudo, Piotr Francuz, Paweł Augustynowicz, Paweł Stróżak

**Affiliations:** The Department of Experimental Psychology, The John Paul II Catholic University of Lublin, Lublin, Poland

**Keywords:** proactive control, reactive control, arousal, approach motivation, P3b, CNV, N2, P3a

## Abstract

A growing body of research has demonstrated that affect modulates cognitive control modes such as proactive and reactive control. Several studies have suggested that positive affect decreases proactive control compared to neutral affect. However, these studies only focused on the valence of affect and often omitted two of its components: arousal and approach motivation. Therefore, we designed the present study to test the hypothesis that cognitive control modes would differ as a function of arousal and approach motivated positive affect. In our study, we used an AX-continuous performance task (AX-CPT), commonly used to examine shifts in proactive and reactive control. We also measured P3b, contingent negative variation (CNV), N2 and P3a components of event-related brain potentials (ERPs) as indicators of the use of cognitive control modes. The findings of the present study demonstrated that approach motivated positive affect modified only the P3b and the CNV without effects on the N2 and P3a components. However, arousal induced by pictures modified P3b, CNV and N2 amplitudes. Specifically, the P3b amplitude was larger, and CNV amplitude was less negative in the high than in the low-approach motivated affect. In contrast, the P3b amplitude was larger and both the CNV and N2 amplitudes more negative in low- compared with high-arousal conditions. These ERP results suggest that approach motivated positive affect enhanced proactive control with no effect on reactive control. However, arousal influenced both proactive and reactive control. High arousal decreased proactive control and increased reactive control compared to low arousal. The present study provides novel insights into the relationship between affect, specifically, arousal and approach motivated positive affect and cognitive control modes. In addition, our results help to explain discrepancies found in previous research.

## Introduction

Cognitive control is defined as a system of processes that maintain the ability to interact with the environment in a goal-driven manner, with flexibility and constantly adapting behavior to the changing environment (Botvinick et al., 2001). Cognitive control is also defined as an emergent process resulting from the dynamic interaction between specialized brain processing systems. Also, this control is possible due to the information of the context in which the task is performed. The context is defined by information about the goals, instructions and requirements relating to the task, as well as information from a previously performed task (Braver et al., 2007). The Dual Mechanism of Control (DMC) framework indicates that cognitive control functions via two distinct operating modes: proactive control and reactive control (Braver et al., 2007; Braver, 2012). Proactive control relates to the active maintenance of contextual information to optimally bias attention, perception and action systems in a goal-driven manner. Reactive control is associated with the retrieval of context information mobilized only as needed, especially after detection of a high interference event (Braver et al., 2007; Braver, 2012). Proactive control is associated with a large number of resources which must be engaged to achieve continuous goal maintenance. As a result, it contributes to limiting the number of goal representations that are the focus of attention and reducing the maintenance of other information (Braver, 2012). It is also connected with the activity of the orbitofrontal–dorsolateral cortex (Braver et al., 2007; Braver, 2012). On the other hand, reactive control is mobilized “in a just-in-time manner” and is, therefore, less resource consuming. The engagement of this control mode is linked to increased anterior cingulate cortex (ACC) activity in response to the detection of interference (Braver, 2012). These types of control can be modulated by several factors, including positive affect (Dreisbach and Goschke, 2004; Dreisbach, 2006; Goschke and Bolte, 2014).

A large number of studies examining the influence of affect on cognitive control within the DMC framework have used the AX-Continuous Performance Task (AX-CPT; Rosvold et al., 1956; Braver and Cohen, 2001; Braver, 2012). This task also requires the ability to update information held in working memory (Goschke and Bolte, 2014). During the task, sequences of letters are shown to the subjects; in each sequence, the first letter is a cue and the second is a probe. There are four possible sequences: (1) AX: the cue is A, and this is followed by the letter X as the probe; (2) AY: the cue is A, and it is followed by any probe other than X; (3) BX: the cue is any letter other than A, and it is followed by the X probe; and (4) BY: the cue is any letter other than A, and it is followed by any probe other than X. The subject’s task is to respond in a specific way (e.g., by pressing the right mouse button) to the probe when it appears as part of an AX sequence. When exposed to the other sequences, the subject is expected to respond differently (e.g., by pressing the left mouse button). This task is an experimental paradigm that establishes context in the form of a specific cue, after which the subject must react to the probe. The sequences are displayed with the following frequencies: AX—70%, AY—10%, BX—10%, BY—10% (Braver, 2012). Therefore, subjects are biased to respond as though for AX sequences when they have AY or BX sequences. Two different error rates and reaction time (calculated for the correct responses) patterns in AY and BX sequences can be observed depending on whether the proactive or reactive control is engaged. Proactive control should create an expectancy for an X probe response following an A cue, which leads to a larger error rates and longer reaction times in the AY sequences. In this context, the longer reaction time may reflect greater interference between the preparatory process followed by the A cue and the response process followed by the Y probe. In the BX sequences, the cue-driven reaction to the probe should lead to fewer error rates and shorter reaction times. This reaction time pattern in BX sequences may occur because the actively maintained contextual information provided by the B cue serve to reduce interference between the preparatory process followed by the B cue and response process followed by the X probe. By contrast, the engagement of reactive control is associated with probe-driven reactions and may lead to fewer error rates and shorter reaction times in AY sequences because the subject does not follow the A cue information when the Y probe is presented. Hence, the subject does not actively maintain contextual information about the A cue and responds on the basis of information about the Y probe which leads to a shortened response time for AY sequences in reactive compared to proactive control. Also, the probe-driven reaction should contribute to more error rates and longer reaction times in the BX sequences. This is related to the fact that the person using reactive control when seeing the X probe is not able to inhibit the learned reaction and change to the less frequent response in the BX sequence. This occurs even though the B cue appears before the X probe. Also, the slower reaction time in the BX sequence in reactive compared to proactive control mode reflects the time taken to engage contextual information about the B cue following X probe presentation (Braver and Cohen, 2001; Braver et al., 2007; Chiew and Braver, 2017).

**Figure 1:**
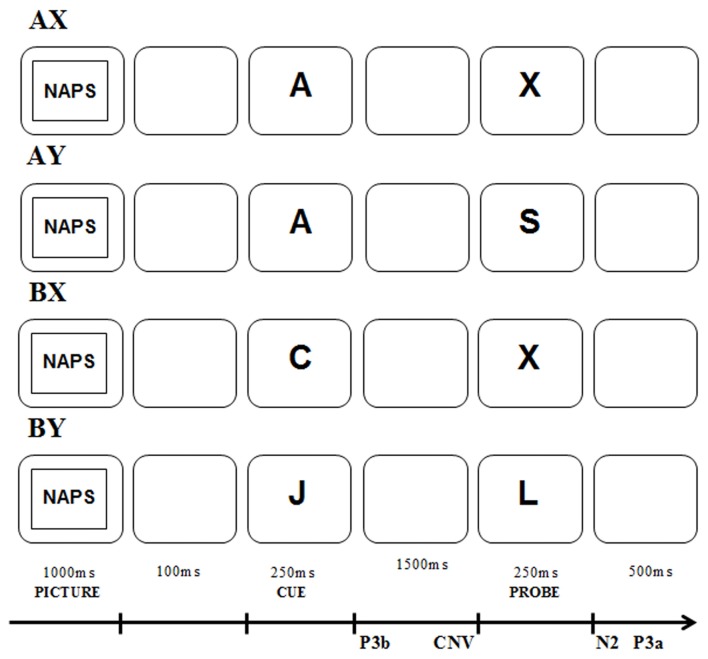
The AX-Continuous Performance Task (AX-CPT) procedure with the four types of sequences occurring in the task.

In addition to behavioral measurements, the AX-CPT method provides reliable indicators of proactive and reactive control using event-related brain potentials (ERPs; see van Wouwe et al., 2011; Morales et al., 2015; Chaillou et al., 2017; Li et al., 2018). Based on previous studies of AX-CPT, the proactive mode of control is assumed to be reflected by P3b analyzed for the cue and contingent negative variation (CNV) analyzed before the probe (see Figure 1). By contrast, reactive control is reflected by N2 and P3a analyzed for the probe (see van Wouwe et al., 2011; Lamm et al., 2013; Morales et al., 2015; Kamijo and Masaki, 2016; Chaillou et al., 2017). P3b is a positive component that reaches its maximum 300–600 ms after stimulus presentation at the Pz electrode (Polich, 2003). This component has multiple functional correlates including context updating, the memory of task-relevant information and target categorization (Polich, 2007). Moreover, a larger P3b is associated with greater context updating and utilization of cue information (Donchin and Coles, 1988; Polich, 2007; Lenartowicz et al., 2010). Therefore, P3b amplitude may reflect enhanced proactive control (van Wouwe et al., 2011). CNV is a slow, surface-negative electrical brain wave occurring in the interval between the presentation of a warning stimulus (e.g., cue) and an imperative stimulus (e.g., probe) to which a motor response is usually required (Tecce, 1972). The CNV component is recorded from the frontal and central electrodes and it is assumed to represent multiple functional correlates including preparing the motor response (Loveless and Sanford, 1975), activation of the attention network (Fan et al., 2007), temporal processing (Mento, 2013), working memory load and response interference (Tecce, 1972; Roth et al., 1975; Gevins et al., 1996; McEvoy et al., 1998). Moreover, a more negative CNV is related to a greater preparatory process for the motor response, particularly where that preparation is preceded by a prior cue that a response is to be prepared (Ruchkin et al., 1995). This may indicate that greater involvement of proactive control is related to more effective task preparation and a larger CNV amplitude. Regarding the reactive control components, the N2 component is a negative component that reaches its maximum 200–400 ms after a conflict situation (Folstein and Van Petten, 2008). Its source of generation is in the medial frontal cortex but is more likely to be in the ACC; (Nieuwenhuis et al., 2003; Folstein and Van Petten, 2008). The ACC, according to the DMC framework is associated with reactive control (Braver et al., 2007; Braver, 2012). The N2 component is usually associated with the monitoring of conflicts relating to the inhibition of incorrect response tendencies caused by either the processing of irrelevant stimuli or choice in the face of competing alternatives (Van Veen and Carter, 2002; Nieuwenhuis et al., 2003). Therefore, it is expected to occur with AY sequences. Larger amplitudes of N2 may reflect stronger conflict detection and may thus be associated with the efficient reactive control. Conversely, P3a is a positive frontoparietal scalp potential with its maximum occurring 300–600 ms after probe presentation. This component reaches a maximum at the FCz electrode (Beste et al., 2011; van Wouwe et al., 2011). The P3a component may be associated with conflict resolution and response inhibition (Bekker et al., 2004; Jonkman, 2006; Polich, 2007; Smith et al., 2008). It is connected with the activity of the ACC (Volpe et al., 2007) that partly supports reactive control (Braver et al., 2007; Braver, 2012). Therefore, larger amplitudes of P3a may reflect enhanced reactive control. It is also expected that its amplitude will be largest for AY sequences as in the case of the N2 component. Concluding, the greater significance of the cues in the proactive control, as opposed to the reactive control, would be expected to elicit a larger cue-related P3b component. Also, the greater expectation of the probe after cue in proactive mode would be expected to elicit a larger CNV compared to the CNV in the reactive control. The greater significance of the probe in the reactive control would be expected to elicit a larger probe-related N2 and probe-related P3a amplitudes here than in the proactive control for AY sequences.

The results of research conducted on the DMC framework (Braver et al., 2007) indicate that positive affect modulates the proactive mode of cognitive control (Dreisbach and Goschke, 2004; Dreisbach, 2006; Fröber and Dreisbach, 2012, 2014). Some researchers have suggested that positive affect is associated with a decrease in proactive control (Dreisbach and Goschke, 2004; Dreisbach, 2006; Fröber and Dreisbach, 2014).

For example, Dreisbach (2006) showed that compared with pictures eliciting neutral and negative affect, those eliciting positive affect reduced error rates in AY trials and increased error rates and reaction times in the BX condition in an AX-CPT. Fröber and Dreisbach (2014) demonstrated that positive affect pictures reduced error rates and reaction times in AY but not in BX sequences. Similarly, van Wouwe et al. (2011) demonstrated that positive rather than neutral affect reduced errors in AY trials but, had no effect on BX sequences. Decreases in error rates in the AY condition may be linked with reduced maintenance of the A cue, which would lead to incorrect preparation for displays of the Y probe. This may result in lower response conflict when the Y probe appears, which may suggest a decrease in proactive control (Braver, 2012). van Wouwe et al. (2011) also showed a more negative probe-related N2 amplitude with neutral affect than with positive affect in AY sequences. van Wouwe et al. (2011) suggested that their results indicated an increase in reactive control and a decrease in proactive control. By contrast, Chiew and Braver (2014) showed increased error rates in AY and decreased error rates in all other sequences in a positive affect block compared with a neutral one. This may indicate the reinforcement of proactive control (Braver, 2012).

In addition to exploring valence, studies have also examined two other dimensions of affect: arousal and approach motivation (see Gable and Harmon-Jones, 2010; Demanet et al., 2011). Arousal is one of the independent affect dimensions defined as a mental activity that can be described along a single dimension ranging from sleep to excitement among other things, in response to a stimulus (Mehrabian and Russell, 1974; Russell and Barrett, 1999). On the other hand, approach motivation is defined as the impulse to go toward stimuli (Lang and Bradley, 2008; Gable and Harmon-Jones, 2010). Arousal is a state of physiological alertness and readiness for action in response to the emergence of an affective stimulus, whereas approach motivation is associated with the action of a person to an affective stimulus (Gable and Harmon-Jones, 2008, 2010). For example, if a person sees a beautiful landscape, such an affective stimulus could generate a low level of arousal but a high motivation to approach it. Furthermore, arousal and approach motivation are connected with different nervous systems. A great deal of recent research suggests that the locus coeruleus-norepinephrine system (LC-NE) is associated with general arousal (Aston-Jones and Cohen, 2005). However, in the case of the approach motivation dimension of positive affect, recent research hypothesizes that the dopamine (DA) system may have a key role in the relationship between motivation and cognitive control (Aarts et al., 2011, 2014; Yee and Braver, 2018).

As regards arousal, it has been shown that low-arousal positive affect reduces cue usage and proactive control, but that high-arousal positive affect increases this type of control. However, no study has found effects for negative affect conditions (Fröber and Dreisbach, 2012). Regarding approach motivation, it has been observed that low-approach motivated positive affect is associated with decreased proactive control and high-approach motivated positive affect enhances proactive control. Specifically, Liu and Xu (2016) showed that error rates were higher in AY sequences and lower in BX sequences in a high-approach motivated positive picture group than in a neutral one. Also, they demonstrated the opposite effect in a low-approach motivated positive picture group than in the a neutral one. More recently, Li et al. (2018) showed that the CNV amplitude analyzed before the probe presentation was larger in a high-approach than in a low-approach condition. They also demonstrated that probe-related P3a was more positive for low than for high-approach motivated positive affect in an AY sequence. However, no effect of approach motivation was found for the probe-related N2 component (Li et al., 2018). This may indicate increasing proactive control in high-approach compared with low-approach motivated positive affect (Gómez et al., 2007; Morales et al., 2015).

To sum up, previous research indicates that the modification of cognitive control is associated with positive affect (Dreisbach and Goschke, 2004; Dreisbach, 2006; Fröber and Dreisbach, 2012; Goschke and Bolte, 2014; Lamm et al., 2013). Positive affect enhances cognitive flexibility in cognitive control and, consequently, impaired maintenance of task-relevant context information and reduced proactive control (Goschke and Bolte, 2014). However, behavioral and electrophysiological findings have suggested that high-approach as opposed to low-approach motivated positive affect enhances proactive control. Furthermore, Fröber and Dreisbach (2012) showed that low-arousal positive affect reduced proactive control whereas high-arousal positive affect increased this type of control. Therefore, previous findings do not provide a coherent explanation of observed differences in the different dimensions of affect. It should be noted that arousal was fully controlled for only in the study by Li et al. (2018), while Fröber and Dreisbach (2012) did not investigate the approach motivation of positive affect. Moreover, the positive affect components arousal and approach motivation have not been compared in any study using the AX-CPT paradigm. Such as comparison could help to understand the discrepancy in studies of the impact of positive affect on cognitive control.

Previous studies have demonstrated different neuronal mechanisms relating to arousal and the approach motivation of affect (Aston-Jones and Cohen, 2005; Demanet et al., 2011; Miller et al., 2013; Braver et al., 2014; Unsworth and Robison, 2017). Therefore, it can be assumed that arousal and the approach motivation of positive affect can independently influence cognitive control. Therefore, the aim of our study was to identify the specific influence of positive affect on proactive and reactive control, considering not only valence but also arousal and the approach motivation of positive affective stimuli simultaneously. On the basis of theoretical discussions, and in accordance with previous studies, we postulated that with high compared with low approach motivation positive affect would be associated with enhanced proactive control (see Liu and Xu, 2016). This would be reflected in the modified amplitudes of the P3b and CNV components. Specifically, we postulated that the P3b amplitude, which is thought to be associated with context updating, would be larger for high- than low-approach motivation. Also, we hypothesize that CNV amplitude, as a functional correlate of preparation for an incoming stimulus would be more negative with high- than low-approach motivation. Considering the Pessoa (2009) model, in which high-arousal stimuli are related to reducing task performance because there is competition between affective stimuli and executive control for attention resources, we expected that high compared to low arousal would be associated with the impaired proactive control. We also hypothesize that both proactive and reactive control would be modified by arousal. The above would be reflected in the P3b, CNV, N2 and P3a component amplitudes. Specifically, we postulated a smaller P3b amplitudes and less negative CNV amplitudes with high compared with low arousal. We also hypothesize that N2 amplitudes, which is thought to be associated with conflict monitoring, would be more negative in the high than in the low arousal. Moreover, we postulated that P3a amplitudes as a functional correlate of conflict resolution will be larger in the high than in the low arousal. To investigate the connection between proactive and reactive control, the electrophysiological method was used, along with high time precision and the AX-CPT paradigm. Considering that individual differences play an important role in modulating affective impact on cognitive control, we used an intra-subject design to control for differences between individuals.

## Materials and Methods

### Participants

The study comprised 25 participants (five men; *M* = 21.32 years, SD = 1.44) who were selected from 748 university students from Lublin. The selection was based on the level of working memory capacity. For this purpose, people performed an Operation Span Task and Symmetry Span Task (Unsworth et al., 2005). The level of working memory capacity was calculated similarly to previous studies (Redick et al., 2012; Redick, 2014). People who achieved the middle results were selected for the study (*M* = 0.05, SD = 0.13) because previous research has shown the difference between people with high and low working memory capacity in proactive control (Redick, 2014; Wiemers and Redick, 2018) The mood of the participants was measured by the Positive and Negative Affect Schedule (PANAS; Watson et al., 1988; Brzozowski, 2010). The participants obtained a mean of 49.96 ± 9.50 in the positive affect scale and a mean of 20.40 ± 5.82 in the negative affect scale. All participants had normal or corrected to normal vision and had no known neurological problems. They volunteered for the study and received a monetary 70 PLN reward (approximately 20 USD). They were informed about the anonymity of the research, and participants gave written consent before the experiment.

This study was carried out in accordance with the recommendations of the Ethical Committee of the Institute of Psychology with written informed consent from all participants. All participants gave written informed consent in accordance with the Declaration of Helsinki. The protocol was approved by the Ethical Committee of the Institute of Psychology of The John Paul II Catholic University of Lublin.

### Procedure

The study applied the paradigm of AX-CPT (Rosvold et al., 1956), using the version proposed by Braver and Cohen (2001) and applied previously in research focusing on the functioning of cognitive control (Braver, 2012). The AX-CPT is a context processing task particularly applied to examine changes in the use of two types of cognitive control: proactive and reactive control. During AX-CPT trials, participants are shown pairs of letters, the first one being a cue, and the second being a probe. There are four possible sequences: (1) AX: the cue is A, and this is followed by the letter X as the probe; (2) AY: the cue is A, and it is followed by any probe other than X; (3) BX: the cue is any letter other than A, and it is followed by the X probe; and (4) BY: the cue is any letter other than A, and it is followed by any probe other than X. The participant’s task is to respond in a specific way (e.g., by pressing the right mouse button) to the probe when it appears as part of an AX sequence. When exposed to the other sequences, the participant is expected to respond differently (e.g., by pressing the left mouse button). The sequences were displayed with the following frequency: AX—70%, AY—10%, BX—10%, BY—10% (Braver, 2012). This frequency is implemented to induce a strong association between the A cue and the X probe in the AX sequence.

The experimental procedure was preceded by a training session, during which the participants practiced the task. At this stage, the participants received feedback on the accuracy of responses. No such information was provided during the experimental trials. Each trial started with the presentation of the picture from the affective picture pool for 1,000 ms, followed by a blank screen shown for 100 ms. Subsequently, the cue letter was displayed for 250 ms. The interval between the contextual cue onset and the probe onset in each trial was 1,750 ms. After this period, the probe was displayed on the screen for 250 ms (see Figure 1). Participants had to press a button each time the probe was presented. In the AX sequence, if the X probe appeared after the A cue, they had to respond with the right button. In other sequences, they had to press the left button. Participants had to press the right button using the right index finger and the left button using the left index finger. To ensure equivalence, halfway through the procedure, the method of responding to the use of the response pad was reversed. All letters were displayed in black color and 28-point Arial font. The procedure does not use letters that are similar in appearance to A or X; for example, K, Y, B, H, R. Affective picture types were organized in separate blocks that were presented randomly to each participant. The experiment began with 40 practice trials. Next, participants performed 600 trials in each affective condition. Each affective condition block was divided into six identical blocks of 100 trials, separated by short breaks in each condition. Stimuli were presented on a 24-inch LCD computer monitor with a display resolution of 1920 × 1080 pixels and a refresh rate of 60 Hz. Participants were seated at a viewing distance of 70 cm from the monitor. The procedure was prepared in the E-Prime software 2.0 (Psychology Software Tools Inc., Sharpsburg, PA, USA).

In order to verify the influence of affect on cognitive control, and in line with previous studies, pictures from a standardized set of affective pictures was used. However, we used the Nencki Affective Picture System (NAPS; Marchewka et al., 2014) instead of the International Affective Picture System (IAPS; Lang et al., 1997). Our choice was influenced by the pictures in the NAPS being divided according to the three dimensions of affect: valence, arousal and approach-avoidance motivation dimensions. In addition, the standardization of pictures was performed on a Polish sample (Marchewka et al., 2014). In these studies, all pictures had positive valence and they were divided into four types: (1) low level of arousal and low level of approach motivation (valence: *M* = 6.52, SD = 0.41; approach-avoidance motivation: *M* = 6.18, SD = 0.32; arousal: *M* = 3.63, SD = 0.29); (2) high level of arousal and high level of approach motivation (valence: *M* = 7.32, SD = 0.22; approach-avoidance motivation: *M* = 7.25, SD = 0.23; arousal: *M* = 5.66, SD = 0.34); (3) high level of arousal and low level of approach motivation (valence: *M* = 6.33, SD = 0.29; approach-avoidance motivation: *M* = 5.88, SD = 0.39; arousal: *M* = 5.42, SD = 0.48); and (4) low level of arousal and high level of approach motivation (valence: *M* = 7.36, SD = 0.39; approach-avoidance motivation: *M* = 7.35, SD = 0.27; arousal: *M* = 2.84, SD = 0.48). The images were displayed at the resolution of 800 × 600. The list of selected pictures can be found in Supplementary Material (Data Sheet 1).

### EEG Recording

Electroencephalograms (EEG) were continuously recorded at a sampling rate of 250 Hz with a high-input impedance amplifier (200 MOhms, EGI Inc., Model: GES 300), using an active electrode system (Brain Products 64-channel actiCAP). The EGI Net Station Version 4.4 was used in the EEG registration. Electrode impedance was maintained below 5 kOhm throughout the experiment. E-Prime 2.0 Professional was used for stimuli presentation.

### ERP Preprocessing

Preprocessing was performed in MATLAB (Mathworks, Natick, MA, USA) using EEGLAB (Delorme and Makeig, 2004). EEG data were re-referenced offline to linked mastoids. As in previous studies (van Wouwe et al., 2011; Chaillou et al., 2017), we used a 0.01–30 Hz offline bandpass filtering for the P3a, P3b and CNV components. For the N2 component, we used a 2–12 Hz offline bandpass filtering, to filter out the P3a component (Donkers et al., 2005). Eye movements and other non-EEG artifacts were corrected by independent component analysis (Delorme et al., 2007). Only epochs with correct responses were kept for averages. The number of trials used for ERP averaging was controlled across conditions; this information is included in a Supplementary Material (Data Sheet 2). Our ERP segmentation and analyses were based on previous ERP studies in which the AX-CPT was used (Beste et al., 2011; van Wouwe et al., 2011; Morales et al., 2015; Chaillou et al., 2017). Epochs were extracted from −200 ms to 800 ms relative to cue or probe onset, with a 200 ms pre-cue or pre-probe baseline respectively. However, the trials for CNV analyses were segmented into 2,200 ms epochs which were extracted from −1,950 ms to 250 ms relative to probe onset with 200 ms pre-cue baseline (see Beste et al., 2011).

The P3b component was analyzed for cue-related potentials. Analyses were conducted over the Pz electrode site because previous studies (Polich, 2007; van Wouwe et al., 2011; Morales et al., 2015) showed that P3b reaches its maximum amplitude at this electrode. The mean amplitude of P3b was calculated in the 450–700 ms time window after cue onset.

On the basis of previous studies (van Wouwe et al., 2011; Morales et al., 2015; Chaillou et al., 2017), the mean amplitude of the CNV was calculated in the time range of 200 to 0 ms before the probe presentation over the Cz electrode. This electrode was chosen because previous studies have indicated that the amplitude is the greatest here (Ruchkin et al., 1995).

The N2 component was analyzed after probe presentation for the probe-related potentials. The analyses were carried out over the FCz electrode because this site is considered to be where the amplitude is greatest (Van Veen and Carter, 2002; van Wouwe et al., 2011; Morales et al., 2015). The mean amplitude of N2 was calculated in the 250–350 ms time window after probe onset.

Also, the P3a component was analyzed for probe-related potentials over the FCz electrode. The mean amplitude of the P3a was calculated in a time range of 350–500 ms after probe onset.

### Data Analysis

For the behavioral data, statistical analyses (three-way mixed ANOVA) were conducted separately for errors and medians of response times (calculated for correct responses) with within-subject factors of APPROACH MOTIVATION (low, high), AROUSAL (lower, higher) and SEQUENCES (AX, AY, BX, BY). Moreover, Proactive Indexes were calculated separately for the medians of response times and error rates according to the formula (AY − BX)/(AY + BX; see Braver et al., 2009; Chiew and Braver, 2014). The result was in the range from −1 to +1. Results approximating +1 reflect the greater involvement of proactive control. The statistical analyses (two-way mixed ANOVA) were similarly conducted for the Proactive Indexes. Simple effects were verified with the Bonferroni *post hoc* test.

For the electrophysiological data, statistical analyses (three-way repeated measures ANOVA) were conducted separately for amplitudes of the CNV and P3b components with within-subject factors of APPROACH MOTIVATION (lower, higher), AROUSAL (lower, higher) and CUES (A, B). For amplitudes of the N2 and P3a components, we performed a three-way repeated measures ANOVA with within-subject factors of APPROACH MOTIVATION (lower, higher), within-subject factors of AROUSAL (lower, higher) and within-subject factors of SEQUENCES (AX, AY, BX, BY). The Bonferroni correction was applied to multiple comparisons. Statistical analysis of the data was performed using the SPSS 21.0 software.

## Results

### Task Performance

**Figure 2:**
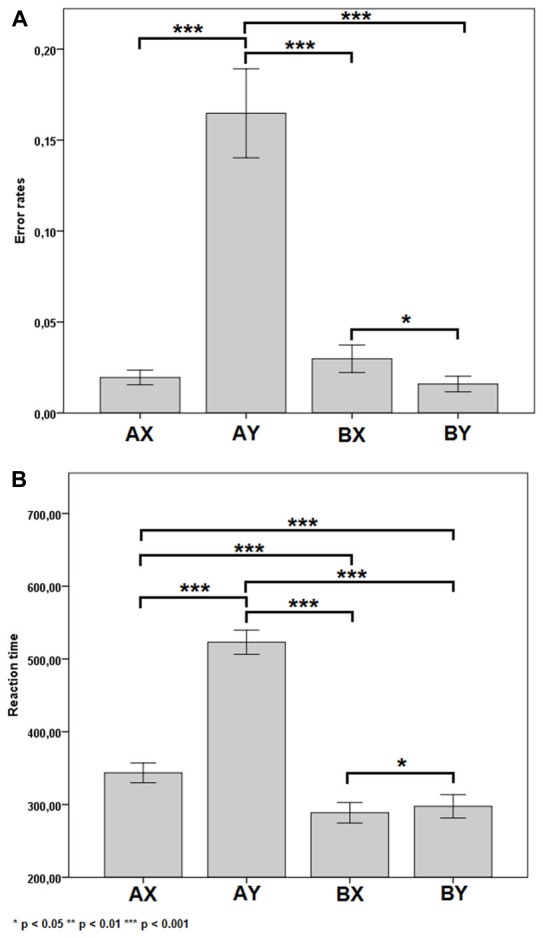
Error rates **(A)** and reaction time **(B)** for each of the four sequences. Error bars represent one standard error of the mean.

For error rates, there was a significant main effect of SEQUENCES (*F*_(3,22)_ = 7.89, *p* < 0.001, $\eta_{\text{p}}^{2}$ = 0.63). The *post hoc* test showed differences in the following pairs of sequences AX-AY (*p* < 0.001), AY-BX (*p* < 0.001), AY-BY (*p* < 0.001), BX-BY (*p* = 0.014). These results are shown in Figure 2A. Other significant main or interactive effects were not yielded (*F* < 1.08, *p* > 0.309).

For reaction times, there was a significant main effect of SEQUENCES (*F*_(3,22)_ = 7.89, *p* < 0.001, $\eta_{\text{p}}^{2}$ = 0.96). The *post hoc* test showed differences in the following pairs of sequences AX-AY (*p* < 0.001), AX-BX (*p* < 0.001), AX-BY (*p* < 0.001), AY-BX (*p* < 0.001), AY-BY (*p* < 0.001) and BX-BY (*p* = 0.045). These results are shown in Figure 2B. Other significant main or interactive effects were not yielded (*F* < 1.28, *p* > 0.305).

For the Proactive Index (error rates), the main effect of APPROACH MOTIVATION (*F*_(1,24)_ = 0.06, *p* = 0.808) and the main effect of AROUSAL (*F*_(1,24)_ = 2.93, *p* = 0.100) were not significant. Also, there was no significant first-order interaction effect of APPROACH MOTIVATION × AROUSAL (*F*_(1,24)_ = 0.19, *p* = 0.287). For Proactive Index (reaction time), there were no significant main effects of APPROACH MOTIVATION (*F*_(1,24)_ = 0.70, *p* = 0.411) or AROUSAL (*F*_(1,24)_ = 0.80, *p* = 0.381). Also, the first-order interaction effect of APPROACH MOTIVATION × AROUSAL was not significant (*F*_(1,24)_ = 1.66, *p* = 0.209).

### ERPs

#### P3b

**Figure 3:**
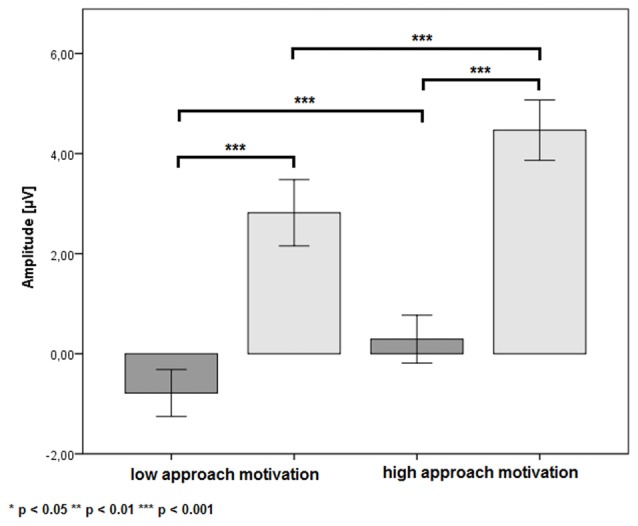
The P3b amplitude as a function of APPROACH MOTIVATION × CUES. Error bars represent one standard error of the mean.

**Figure 4:**
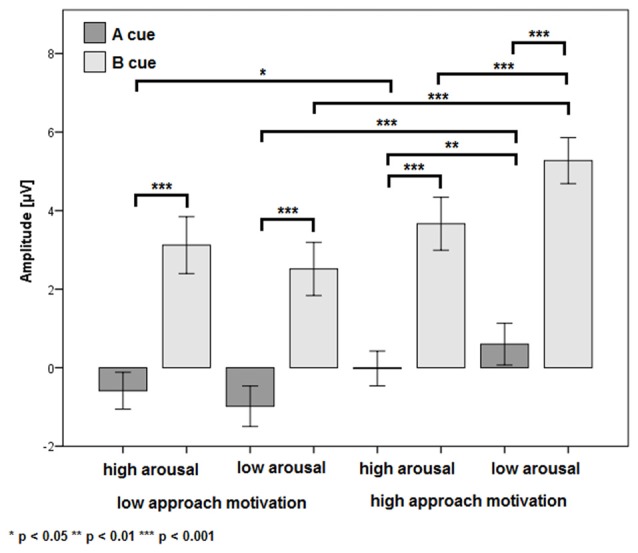
The P3b amplitude as a function of AROUSAL × APPROACH MOTIVATION × CUES. Error bars represent one standard error of the mean.

**Figure 5:**
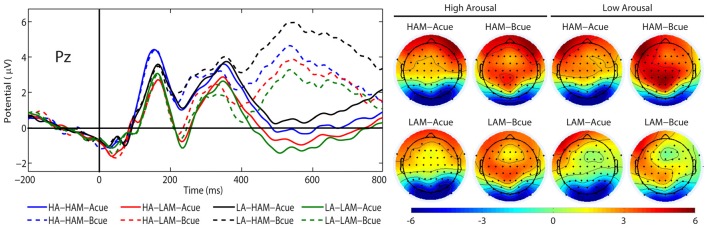
Grand average event-related brain potentials (ERPs) (P3b) and topographical maps elicited by the A cue (solid lines) and the B cue (dashed lines) at Pz, separately for low (LA) and high (HA) arousal and low (LAM) and high (HAM) motivated positive affect.

There was a significant main effect of the factor CUES (*F*_(1,24)_ = 71.38, *p* < 0.001, *η*^2^ = 0.75). The P3b amplitude was more positive in the B cue condition (*M* = 3.64 μV, SE = 0.62 μV) than in the A cue condition (*M* = −0.25 μV, SE = 0.47 μV). Also, the main effect of the factor APPROACH MOTIVATION was significant (*F*_(1,24)_ = 71.38, *p* < 0.001, *η*^2^ = 0.74). The P3b amplitude was larger in the high-approach motivation condition (*M* = 2.38 μV, SE = 0.50 μV) than in the low-approach motivation condition (*M* = 1.02 μV, SE = 0.51 μV). There was a significant first-order interaction effect for APPROACH MOTIVATION × AROUSAL (*F*_(1,24)_ = 19.50, *p* < 0.001, *η*^2^ = 0.45). The effect showed the different patterns of P3b amplitude in the high- and low-approach motivation condition. Based on the *post hoc* test, the difference between low and high arousal has been shown in the high-approach motivation condition (*p* < 0.001). Specifically, the P3b amplitude was smaller in high arousal (*M* = 1.82 μV, SE = 0.51 μV) than low arousal (*M* = 2.94 μV, SE = 0.52 μV). The analogous difference was not observed in the low-approach motivation condition (*p* = 0.170). Furthermore, the *post hoc* test showed that the P3b amplitude was larger in high-approach motivation (*M* = 1.82 μV, SE = 0.51 μV) than low-approach motivation (*M* = 1.27 μV, SE = 0.54 μV) in high-arousal conditions (*p* = 0.029). The analogous difference was observed in the low-arousal condition (*p* < 0.001). The first-order interaction effect for APPROACH MOTIVATION × CUES was significant (*F*_(1,24)_ = 6.44, *p* = 0.018, *η*^2^ = 0.21). The *post hoc* test showed that difference between the A cue and the B cue was significant in high- (*p* < 0.001) and low-approach motivation conditions (*p* < 0.001). We also observed differences between low- and high-approach motivation in the A cue (*p* < 0.001) and the B cue (*p* < 0.001) conditions. The results are shown in Figure 3. There was a significant second-order interaction effect for AROUSAL × APPROACH MOTIVATION × CUES (*F*_(1,24)_ = 6.98, *p* = 0.014, *η*^2^ = 0.23). The results are shown in Figure 4. There was no significant main effect for the factor AROUSAL (*F*_(1,24)_ = 1.56, *p* = 0.223) or the first-order interaction effect AROUSAL × CUES (*F*_(1,24)_ = 2.32, *p* = 0.141). Figure 5 illustrates the average ERP waveforms after the A and B cues for each condition.

#### CNV

**Figure 6:**
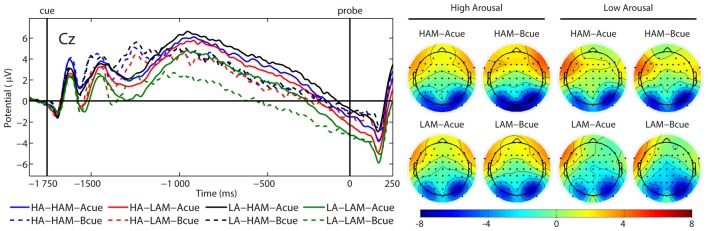
Grand average (ERPs) contingent negative variation (CNV) and topographical maps elicited by the A cue (solid lines) and the B cue (dashed lines) at Cz, separately for low (LA) and high (HA) arousal and low (LAM) and high (HAM) motivated positive affect.

There was a significant main effect of factor APPROACH MOTIVATION (*F*_(1,24)_ = 19.41, *p* < 0.001, *η*^2^ = 0.45). The CNV amplitude was more negative in the low condition (*M* = −1.64 μV, SE = 0.61 μV) than in the high-approach motivation condition (*M* = −0.21 μV, SE = 0.68 μV). Also, there was a significant first-order interaction effect for APPROACH MOTIVATION × AROUSAL (*F*_(1,24)_ = 8.93, *p* = 0.006, *η*^2^ = 0.27). The effect showed the different patterns of CNV amplitude in the high- and low-approach motivation condition. Based on the *post hoc* test, the difference between low and high arousal has been shown in the low-approach motivation condition (*p* = 0.004). Specifically, the CNV amplitude was more negative in the low-arousal (*M* = −2.36 μV, SE = 0.69 μV) than in the high-arousal (*M* = −0.91 μV, SE = 0.60 μV). The analogous difference was not observed in the high-approach motivation condition (*p* = 0.451). In addition, different patterns of CNV amplitude were showed in in the high- and low-arousal condition. Based on the *post hoc* test, the difference between the high- and low-approach motivation has been shown in the low-arousal condition (*p* < 0.001). Concretely, the CNV amplitude was more negative in the low- (*M* = −2.36 μV, SE = 0.69 μV) than in the high-approach motivation (*M* = −0.01 μV, SE = 0.71 μV). No analogous difference was observed in the high-arousal condition (*p* = 0.271). There was a significant first-order interaction effect for AROUSAL × CUES (*F*_(1,24)_ = 6.29, *p* = 0.019, *η*^2^ = 0.21). However, no simple effects were significant. Other significant main or interactive effects were not yielded (*F* < 1.89, *p* > 0.182). Figure 6 illustrates grand average ERP waveforms after the A and B cues for each condition.

#### N2

**Figure 7:**
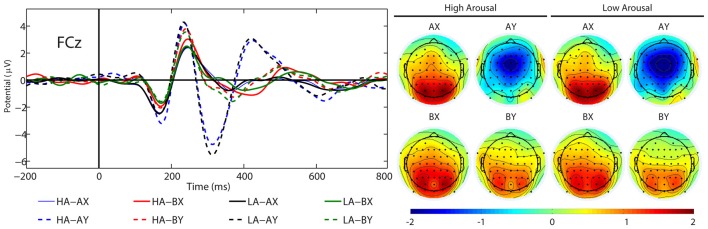
Grand average ERPs (N2) and topographical maps elicited by the AX, AY, BX and BY sequences at FCz, separately for low (LA) and high (HA) arousal.

The main effect of SEQUENCES was statistically significant (*F*_(3,22)_ = 18.98, *p* = 0.442, *η*^2^ = 0.72). Analysis using the *post hoc* test showed that the N2 amplitude in the AY sequence (*M* = −2.78 μV, SE = 0.51 μV) was larger than in the AX (*M* = 0.73 μV, SE = 0.25 μV, *p* < 0.001), BX (*M* = 0.74 μV, SE = 0.26 μV, *p* < 0.001) and BY (*M* = 0.27 μV, SE = 0.32 μV, *p* < 0.001) sequences. Also, there was a significant main effect of the factor AROUSAL (*F*_(1,24)_ = 6.81, *p* = 0.015, *η*^2^ = 0.22). The N2 amplitude was less negative in high (*M* = −0.14 μV, SE = 0.25 μV) than in low levels of arousal condition (*M* = −0.37 μV, SE = 0.29 μV). It should be noted that simple effect showed that the difference between these conditions occurs only in the AY sequence. Other significant main or interactive effects were not yielded (*F* < 1.25, *p* > 0.315). Figure 7 illustrates the grand average ERP waveforms after probe presentation in the AX, AY, BX and BY sequences for low and high arousal.

#### P3a

**Figure 8:**
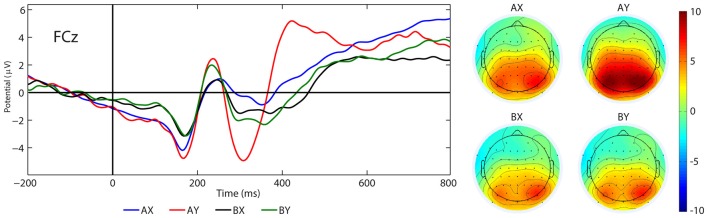
Grand average ERPs (P3a) and topographical maps elicited by the AX, AY, BX and BY sequences at FCz.

There was a significant main effect of the factor SEQUENCES (*F*_(3,22)_ = 9.75, *p* < 0.001, *η*^2^ = 0.57). The *post hoc* test showed that the P3a amplitude in the AY sequence (*M* = 3.74 μV, SE = 0.97 μV) was larger than for the AX (*M* = 1.09 μV, SE = 1.03 μV, *p* = 0.003), BX (*M* = −0.32 μV, SE = 0.91 μV, *p* < 0.001) and BY (*M* = 0.30 μV, SE = 0.95 μV, *p* < 0.001) sequences. Other significant main or interactive effects were not yielded (*F* < 3.22, *p* > 0.086). Figure 8 illustrates the grand average ERP waveforms after probe presentation in the AX, AY, BX and BY sequences.

## Discussion

The current study investigated how approach motivation, positive affect and arousal induced by pictures had effects on cognitive control, particularly in the field of proactive and reactive control. Similar to previous studies (Liu and Xu, 2016; Li et al., 2018), we hypothesized that high-compared to low-approach motivated positive affect would be associated with the enhanced proactive control. Also, based on the Pessoa (2009) model and LC-NE functioning (Aston-Jones and Cohen, 2005), we postulated that high compared to low arousal would be associated with reduced proactive control and enhanced reactive control. We examined this hypothesis using the AX-CPT paradigm and electrophysiological method to measure ERP components associated with both cognitive control modes. The results mostly confirmed our hypothesis and demonstrated that high-approach motivated positive affect enhanced proactive control without any effect on reactive control. Also, they showed that high arousal induced by pictures reduced proactive control and reinforced reactive control. We first discuss the behavioral results and the effect of the analyzed factors on proactive control. We then present the influence of arousal and approach motivation on reactive control.

Our results showed no effect of any affect dimension on any AX-CPT sequences at the behavioral level. We only showed the standard effect of AY sequences. Specifically, error rates for this sequence were the highest and reaction times the longest of all sequences (see Braver et al., 2007; Braver, 2012; Cooper et al., 2017). However, our findings are similar to the behavioral results found in previous ERP studies examining the impact of affect on cognitive control in the AX-CPT paradigm (Chaillou et al., 2017; Li et al., 2018). A possible explanation for this situation could be the greater number of task trials in the ERP research than in the behavioral study. This can lead to a practice effect on behavioral performance (see Braver et al., 2009).

Regarding approach motivation, we observed that the P3b amplitude was larger in the high- than in the low-approach motivated positive affect condition. Nor was this effect dependent on the level of arousal induced by the pictures. Also, this difference in P3b amplitude was evident for both the A and B cues, and the P3b amplitude being more positive in the B cue than in the A cue. Thus, this pattern may indicate that high-approach motivated, positive affect is associated with enhanced context updating and larger utilization of cue information both in the A and B cues (see Donchin and Coles, 1988; Polich, 2007; Lenartowicz et al., 2010). This result suggests that high-approach motivated positive affect reinforces of proactive control, while low-approach motivated positive affect may lead to a decrease in this mode of cognitive control. This supposition is in line with earlier studies that have shown the difference between high- and low-approach motivated positive affect in relation to cognitive flexibility and stability. Low-approach motivated positive affect enhanced cognitive flexibility and distractibility, whereas high-approach motivated positive affect increased perseverance and reduced distractibility (Liu and Wang, 2014; Liu and Xu, 2016). Greater flexibility may be associated with decrease proactive control (Dreisbach and Goschke, 2004; Dreisbach, 2006). Hence, compared with high approach motivation, low approach motivation contributes to decreasing proactive control. This is in line with our findings for the P3b component.

Concerning arousal induced by the picture, we observed that the P3b amplitude was smaller for high than for low arousal in the high-approach motivation condition. This difference in P3b amplitude was evident only for the B cue. Hence, this pattern may indicate that low arousal is associated with enhanced context updating and larger utilization of B cue information (see Donchin and Coles, 1988; Polich, 2007; Lenartowicz et al., 2010). According to the DMC framework, proactive control engages a large number of resources to maintain contextual information. This reduces the number of goal representations in the focus of attention (Braver, 2012). Pessoa (2009, 2017) postulated that high-arousal stimuli are related to the greater rivalry between affective stimuli and executive control for attention resources. Previous studies have shown that emotional arousal reduces activity in the cortical regions involved in cognitive control process and enhances activity in the cortical regions involved in the emotion processes (Hart et al., 2010). Also, Pessoa et al. (2012) showed that high-arousal emotional stimuli as stop signals lead to worsened response inhibition, whereas low-arousal stop signals enhance inhibition. Also, Kuhbandner and Zehetleitner (2011) showed that the attentional selection of cues in a high-arousal situation is stimulus-driven salience of a stimulus but does not have goal-driven task relevance. Taken together, these findings suggest that proactive control requires attention resources to maintain goal-irrelevant information. However, these resources may be taken away by high emotional arousal. High compared to low arousal induced by pictures may lead to a reduction in proactive control, which would be reflected in the P3b amplitude.

Contrary to our hypothesis, our results showed that CNV amplitude was more negative in the low- than in the high-approach motivated positive affect condition. The CNV component is thought to be associated with the preparatory process for the motor response. In particular where the preparation of a motor response is preceded by a prior cue that the response is to be prepared (Ruchkin et al., 1995). This may indicate that greater engagement of proactive control is related to more effective task preparation and larger CNV amplitude. Thus, this pattern may indicate that low-approach motivated positive affect is associated with stronger response preparation processes than the high-approach motivated positive affect. Hence, the greater CNV amplitude may reflect increases in proactive control (Li et al., 2018). In line with this account, low-approach motivated positive affect may lead to an increase in this cognitive control mode. This supposition is in contradiction to our hypothesis and P3b results. However, other research has shown a smaller CNV component in conditions requiring active maintenance of a task goal in the working memory than an anticipated simple motor reaction (Vanderhasselt et al., 2014). This is in line with previous studies indicating that the CNV amplitude reduces with increasing working memory load or increasing response interference (Tecce, 1972; Roth et al., 1975; Gevins et al., 1996; McEvoy et al., 1998). Considering that the CNV component may reflect the working memory load related to maintaining information about the cue (see Onoda et al., 2004), our results may have another interpretation. In this regard, greater active maintenance of goal-relevant information should lead to greater working memory load. This load should be reflected in the CNV amplitude. Specifically, a less negative CNV amplitude may reflect a greater working memory load. According to the DMC framework, proactive control engages large resources which are involved in the active maintenance of goal-relevant information control (Braver, 2012; Chiew and Braver, 2017) so it may lead to greater working memory load. In this context, decreased CNV amplitude may be an indicator of increased proactive control. In line with this explanation, low-approach motivated positive affect may decrease proactive control whereas high-approach motivated positive affect may increase this cognitive control mode. However, this interpretation requires further research and should be considered with caution. In addition, it should be taken into account that the slow positive shift may overlap CNV activity and may influence the results obtained (Curry, 1984). All the more so because the CNV reflects the confounding of attention to the upcoming stimulus and preparation for the response, which take place simultaneously (Brunia et al., 2012). This could be one of several possible explanations for the different results obtained in previous studies using the AX-CPT paradigm (see van Wouwe et al., 2011; Kamijo and Masaki, 2016; Chaillou et al., 2017; Li et al., 2018).

Concerning arousal induced by the picture, we demonstrated that the CNV amplitude was more negative in the low than in the high arousal condition in the low-approach motivated positive affect condition. One possible explanation for this relates to the fact that a larger CNV amplitude may reflect more effective preparation for the motor response and consequently greater engagement of proactive control. In this regard, low compared with high arousal may lead to an increase in proactive control which is in line with our hypothesis. Other possible explanation relates to the fact that our result may be associated with a greater working memory load in high arousal induced by the picture. However, in this situation, the working memory load may be related to the allocation of resources to emotion processing. This would be in line with the model proposed by Pessoa (2009, 2017). Thus, this pattern may indicate that active maintenance of the task goal is easier in a low-than in a high-arousal condition. Additionally, proactive control is related to the active maintenance of context representations and goal-driven behavior (Braver, 2012; Chiew and Braver, 2017). Taken together, the findings suggest that proactive control may be more supported by low than by high arousal which is also in line with our hypothesis. However, this explanation requires further research and should be considered carefully.

We found no approach motivation effect on the N2 component. Our findings seem to be in line with previous findings by Li et al. (2018), who did not show a difference between high- and low-approach motivated positive affect with N2 amplitude. In addition, Chaillou et al. (2017) found no difference between positive and neutral affect in the N2 component. However, our results demonstrated that the N2 amplitude was more negative in low than in high levels of arousal. The results of previous studies have shown that the N2 component is a reflection of conflict monitoring related to either the inhibition of incorrect response tendencies caused by irrelevant stimuli or the choice of reaction in the face of competing alternatives (Van Veen and Carter, 2002; Nieuwenhuis et al., 2003). Hence, this pattern may indicate greater cognitive conflict and enhanced reactive control in the low- than in the high-arousal condition. However, this conflict occurs only in the AY sequence. Considering that the N2 component may reflect the actual control process (see Folstein and Van Petten, 2008), our results may have another interpretation. In this situation, greater activation of goal information (the A cue) should lead to greater interference between goal representation and the probe in the AY sequence. This interference should be reflected in the N2 amplitude. Specifically, a more negative N2 amplitude may indicate greater interference in the AY sequence. According to the DMC framework, greater interference in the AY sequence is related to enhanced proactive control (Braver, 2012; Chiew and Braver, 2017). Hence, the more negative N2 amplitude in the AY sequence may reflect increased proactive control or decreased reactive control. Considering this interpretation, our results may indicate a reduction in reactive control or enhanced proactive control in low- than high-arousal conditions. This explanation is in line with previous studies showing a more negative N2 amplitude in neutral affect compared with positive affect in AY sequences (van Wouwe et al., 2011).

We did not find an approach motivation effect, and we did not find an arousal effect on the P3a component. We only observed the typical effects according to sequence type: the P3a amplitude was more positive in the AY sequences compared to the AX, BX and BY sequences (Morales et al., 2015; Chaillou et al., 2017; Li et al., 2018). This result may indicate that approach motivation and arousal do not impact on proactive control or reactive control. However, previous research have postulated that P3a is related to focal attention and working memory mediated by DA activity (see Polich, 2007). On the other hand, other study results have suggested a relationship between P3a and the LC-NE system (see Howells et al., 2012). Therefore, the manipulation of approach motivation and arousal introduced by us could increase the variance associated with the activation of different neuronal systems.

Our results showed that approach motivation only modified the P3b and CNV components, without effects on the N2 or P3a components. This may indicate that approach motivated positive affect only modulates proactive control. Specifically, high-approach motivated positive affect enhanced proactive control, whereas low-approach motivated positive affect reduced proactive control. Our findings seem to be in line with those of other studies (Liu and Wang, 2014; Liu and Xu, 2016). Also, we showed that arousal induced by pictures modified P3b, CNV and N2 amplitudes. Considering that P3b and CNV reflect a change in proactive control and N2 reflects variation in reactive control, it can be assumed that arousal influences both types of control. Specifically, low arousal induced by pictures promoted increased proactive control and reduced reactive control. On the other hand, high arousal induced by pictures is related to reduced proactive control and enhanced reactive control. However, the results of Fröber and Dreisbach (2012) are contrary to our findings. It should be noted that Fröber and Dreisbach (2012) did not control approach motivated positive affect. In their study, the high-arousal picture displayed group sport and adventure pictures, and the low-arousal pictures showed babies and families. In our study, high-arousal and high-approach motivated pictures showed group and individual sport, whereas low-arousal and low-approach motivated pictures presented the faces of children and other people. Also, Fröber and Dreisbach (2012) demonstrated that affect modulates only proactive control. However, our findings indicate that approach motivation influences proactive control whereas arousal influences both proactive and reactive control. Therefore, we may very cautiously suppose that the effect observed by Fröber and Dreisbach (2012) may have been driven by approach motivated positive affect, not by arousal. However, further research is required to explain the observed differences.

In conclusion, our study is one of the first to explore simultaneously approach motivated positive affect and arousal influence on cognitive control in an AX-CPT paradigm. Our results showed that approach motivated positive affect modulated proactive control with no effect on reactive control. However, arousal influenced both proactive and reactive control. These results may indicate that approach motivated positive affect may be conducive to more precise preparation of one’s actions through available information. However, arousal may modify the control mechanism as a result of a cognitive conflict that may contribute to changing the goals of the action (see Cohen et al., 2004). Our findings may contribute to a better understanding of the relationship between affect and cognitive control. However, further research is needed to explain the observed results better. In particular, taking into account the DMC framework, it is important to consider how arousal impacts on the maintaining and changing of information about the goal of the action in the situation of cognitive conflict and the change of goal representation. Also to be tested, taking into account the relationship between arousal and working memory capacity (Unsworth and Robison, 2017), is whether our results would be different for people with high vs. low working memory capacity.

## Author Contributions

AC and PF: substantial contributions to the conception of the work and substantial contributions to the design of the work. AC, PA and PS: the acquisition, analysis, or interpretation of data for the work. AC and PF: drafting the work. AC, PF, PA and PS: revising the work critically for important intellectual content, final approval of the version to be published and agreement to be accountable for all aspects of the work in ensuring that questions related to the accuracy or integrity of any part of the work are appropriately investigated and resolved.

## Conflict of Interest Statement

The authors declare that the research was conducted in the absence of any commercial or financial relationships that could be construed as a potential conflict of interest.
