# Supplementary material for: The Effects of Arousal and Approach Motivated Positive Affect on Cognitive Control. An ERP Study
Source: Front Hum Neurosci. 2018 Aug 31;12:320. doi: 10.3389/fnhum.2018.00320 (PMC6128242; doi:10.3389/fnhum.2018.00320)
Supplement: Supplementary file 1 [file Data_Sheet_1.pdf]

Numbers of affective picture stimuli:

1) low level of arousal and low level of approach motivation:
Faces_203_h
Faces_115_h
Faces_249_v
Faces_052_h
Faces_243_v
Faces_181_v
Faces_214_h
Faces_075_v
Faces_195_v
Faces_199_v

2) high level of arousal and high level of approach motivation:
People_180_h
People_096_h
People_196_h
People_175_h
People_193_h
People_030_h
People_130_h
People_183_h
People_160_h
People_189_h

3) high level of arousal and low level of approach motivation:
Animals_198_h
Animals_171_h
Animals_055_h
Animals_130_h
Animals_122_h
Animals_197_h
Animals_215_v
Animals_040_v
Animals_002_v
Animals_144_h

4) low level of arousal and high level of approach motivation:
Landscapes_049_h
Landscapes_109_h
Landscapes_097_v
Landscapes_048_h
Landscapes_105_v
Landscapes_104_h
Landscapes_141_h
Landscapes_152_h
Landscapes_122_v
Landscapes_050_h
