# Supplementary material for: The Effects of Arousal and Approach Motivated Positive Affect on Cognitive Control. An ERP Study
Source: Front Hum Neurosci. 2018 Aug 31;12:320. doi: 10.3389/fnhum.2018.00320 (PMC6128242; doi:10.3389/fnhum.2018.00320)
Supplement: Supplementary file 2 [file Data_Sheet_2.pdf]

Information on the number of single trials used for analysis of different conditions:

P3b

HA-HAM-Acue	M = 360.00	(SD = 85.78; range: 200 - 462)
HA-HAM-Bcue	M = 93.60	(SD = 18.58; range: 60 - 119)
HA-LAM-Acue	M = 363.40	(SD = 78.86; range: 177 - 460)
HA-LAM-Bcue	M = 95.08	(SD = 17.07; range: 47 - 118)
LA-HAM-Acue	M = 366.96	(SD = 69.16; range: 206 – 467)
LA-HAM-Bcue	M = 95.44	(SD = 14.33; range: 52 - 119)
LA-LAM-Acue	M = 366.92	(SD = 79.73; range: 136 - 469)
LA-LAM-Bcue	M = 95.64	(SD = 18.82; range: 40 – 120)

CNV

HA-HAM-Acue	M = 375.32	(SD = 66.86; range: 234 – 464)
HA-HAM-Bcue	M = 94.36	(SD = 15.91; range: 59 – 120)
HA-LAM-Acue	M = 371.48	(SD = 65.85; range: 225 – 464)
HA-LAM-Bcue	M = 92.76	(SD = 17.28; range: 49 – 117)
LA-HAM-Acue	M = 371.4	(SD = 59.41; range: 230 – 463)
LA-HAM-Bcue	M = 93.12	(SD = 13.65; range: 63 – 119)
LA-LAM-Acue	M = 374.48	(SD = 68.81; range: 176 – 465)
LA-LAM-Bcue	M = 94.12	(SD = 17.70; range: 51 – 120)

N2

HA-LAM-AX	M = 360.68	(SD = 41.38; range: 275 – 418)
HA-LAM-AY	M = 46.76	(SD = 10.38; range: 24 - 59)
HA-LAM-BX	M = 50.96	(SD = 5.54; range: 40 – 59)
HA-LAM-BY	M = 52.12	(SD = 4.69; range: 42 – 60)
LA-LAM-AX	M = 366.6	(SD = 46.51; range: 212 – 418)
LA-LAM-AY	M = 46.92	(SD = 9.47; range: 24 – 60)
LA-LAM-BX	M = 52.80	(SD = 6.75; range: 35 – 60)
LA-LAM-BY	M = 52.32	(SD = 5.56; range: 40 – 60)
LA-HAM-AX	M = 363.12	(SD = 37.61; range: 288 – 416)
LA-HAM-AY	M = 46.24	(SD = 9.51; range: 19 – 56)
LA-HAM-BX	M = 51.84	(SD = 4.64; range: 42 – 59)
LA-HAM-BY	M = 52.40	(SD = 4.55; range: 42 – 59)
HA-HAM-AX	M = 366.24	(SD = 45.23; range: 261 – 416)
HA-HAM-AY	M = 47.44	(SD = 7.48; range: 32 – 58)
HA-HAM-BX	M = 51.76	(SD = 5.53; range: 39 – 60)
HA-HAM-BY	M = 53.12	(SD = 4.62; range: 39 – 59)

P3a

HA-LAM-AX	M = 313,20	(SD = 69,38; range: 134 – 399)
HA-LAM-AY	M = 39,44	(SD = 12,35; range: 18 – 57)
HA-LAM-BX	M = 45,92	(SD = 9,34; range: 27 – 59)
HA-LAM-BY	M = 46,44	(SD = 8,69; range: 25 – 58)

LA-LAM-AX	M = 323,24	(SD = 69,11; range: 108 – 411)
LA-LAM-AY	M = 40,36	(SD = 11,59; range: 23 – 59)
LA-LAM-BX	M = 48,24	(SD = 9,30; range: 20 – 59)
LA-LAM-BY	M = 47,36	(SD = 10,17; range: 16 – 59)
LA-HAM-AX	M = 317,20	(SD = 63,37; range: 143 – 401)
LA-HAM-AY	M = 39,32	(SD = 11,27; range: 12 – 54)
LA-HAM-BX	M = 46,56	(SD = 7,34; range: 29 – 58)
LA-HAM-BY	M = 46,36	(SD = 8,83; range: 23 – 58)
HA-HAM-AX	M = 313,32	(SD = 79,67; range: 155 – 410)
HA-HAM-AY	M = 39,32	(SD = 10,05; range: 19 – 58)
HA-HAM-BX	M = 47,12	(SD = 8,10; range: 28 – 59)
HA-HAM-BY	M = 47,88	(SD = 8,20; range: 29 – 58)

HA – high arousal
LA – low arousal
HAM – high-approach motivation
LAM – low-approach motivation
